# ^18^F-Fluoroazomycin Arabinoside (FAZA) PET/MR as a Biomarker of Hypoxia in Rectal Cancer: A Pilot Study

**DOI:** 10.3390/tomography10090102

**Published:** 2024-08-30

**Authors:** Ur Metser, Andres Kohan, Catherine O’Brien, Rebecca K. S. Wong, Claudia Ortega, Patrick Veit-Haibach, Brandon Driscoll, Ivan Yeung, Adam Farag

**Affiliations:** 1 University Medical Imaging Toronto, University Health Network, Sinai Health Systems, Women’s College Hospital, University of Toronto, Toronto, ON M5G 2N2, Canadaclaudia.ortega@uhn.ca (C.O.); patrick.veit-haibach@uhn.ca (P.V.-H.); adam.farag@uhn.ca (A.F.); 2 Department of Surgery, University Health Network, Toronto, ON M5G 2C4, Canada; 3 Radiation Medicine Program, Princess Margaret Cancer Centre, University Health Network, Toronto, ON M5G 2M9, Canada; 4 Quantitative Imaging for Personalized Cancer Medicine, Techna Institute, University Health Network, Toronto, ON M5G 2C4, Canada

**Keywords:** hypoxia, rectal cancer, PET/MRI, ^18^F-FAZA

## Abstract

Tumor hypoxia is a negative prognostic factor in many tumors and is predictive of metastatic spread and poor responsiveness to both chemotherapy and radiotherapy. **Purpose:** To assess the feasibility of using ^18^F-Fluoroazomycin arabinoside (FAZA) PET/MR to image tumor hypoxia in patients with locally advanced rectal cancer (LARC) prior to and following neoadjuvant chemoradiotherapy (nCRT). The secondary objective was to compare different reference tissues and thresholds for tumor hypoxia quantification. **Patients and Methods:** Eight patients with histologically proven LARC were included. All patients underwent ^18^F-FAZA PET/MR prior to initiation of nCRT, four of whom also had a second scan following completion of nCRT and prior to surgery. Tumors were segmented using T_2_-weighted MR. Each voxel within the segmented tumor was defined as hypoxic or oxic using thresholds derived from various references: ×1.0 or ×1.2 SUVmean of blood pool [BP] or left ventricle [LV] and SUVmean +3SD for gluteus maximus. Correlation coefficient (CoC) between HF and tumor SUVmax/reference SUVmean TRR for the various thresholds was calculated. Hypoxic fraction (HF), defined as the % hypoxic voxels within the tumor volume was calculated for each reference/threshold. **Results:** For all cases, baseline and follow-up, the CoCs for gluteus maximus and for BP and LV (×1.0) were 0.241, 0.344, and 0.499, respectively, and HFs were (median; range) 16.6% (2.4–33.8), 36.8% (0.3–72.9), and 30.7% (0.8–55.5), respectively. For a threshold of ×1.2, the CoCs for BP and LV as references were 0.611 and 0.838, respectively, and HFs were (median; range) 10.4% (0–47.6), and 4.3% (0–20.1%), respectively. The change in HF following nCRT ranged from (−18.9%) to (+54%). **Conclusions:** Imaging of hypoxia in LARC with ^18^F-FAZA PET/MR is feasible. Blood pool as measured in the LV appears to be the most reliable reference for calculating the HF. There is a wide range of HF and variable change in HF before and after nCRT.

## 1. Introduction

Colorectal cancer is the second cause of cancer deaths worldwide and is the leading cause of death in men under 50 years of age [1]. Rectal cancer comprises approximately 30% of all colorectal cancers. Early-stage tumors are treated with upfront surgery, while locally advanced rectal cancers (LARC) require multimodal therapy often consisting of neoadjuvant chemoradiotherapy and then total mesorectal excision [2,3,4,5,6]. In LARC, neoadjuvant chemoradiotherapy results in improved local tumor control and overall survival, especially in those achieving complete response to therapy. Multiple factors can impact response to neoadjuvant therapy, including epigenetic factors and tumor hypoxia, which is more prevalent in advanced tumors [7,8]. Hypoxia occurs in solid tumors in areas where the consumption of oxygen outpaces the delivery from the vascular system. It is a negative prognostic factor in many tumors and is predictive of metastatic spread and poor responsiveness to both chemotherapy and radiotherapy [9,10,11,12]. Hypoxia limits radiation-induced DNA damage by reducing DNA double-strand breaks, the most lethal form of DNA damage [9]. Hypoxia-induced chemoresistance is multifactorial and includes decreased drug activity with reduced oxygen, pH changes, reduced proliferation, induction of prosurvival gene expression, and difficulty in drug diffusion from vasculature [9]. Specific to colorectal cancer, increased activity of transcription factor hypoxia inducible factor 1α (HIF-1a) reduces the efficacy of 5-Fluorouracil, the backbone of chemotherapy in colorectal cancer and a radiosensitizer in the context of concurrent chemoradiotherapy (CRT) [10]. Although a direct correlation to response to CRT has not been clinically proven, studies have demonstrated a poorer disease-specific survival in patients with rectal cancer expressing HIF-1a [12]. Furthermore, CD 133+ cancer stem-like cells (cCSCs) have been shown to be protected in the hypoxic regions of colorectal cancers, providing a permissive environment for tumor recurrence [12]. Hence, there is a rationale for the identification of hypoxic rectal cancers for prognostication and stratification of response to chemotherapy.

Several tracers have been used to noninvasively image tumor hypoxia, including ^18^F-1-α-D-[5-fluoro-5-deoxyarabinofuranosyl]-2-nitroimidazole (^18^F-FAZA), a second-generation 2-nitroimidazole with lipophilicity which enables it to enter cells. When in a hypoxic environment, it is reversibly reduced to reactive oxygen radicals and trapped within cells, enabling imaging and quantification of hypoxia [13,14]. Pimonidazole, an extrinsic marker of hypoxia, is a 2-nitroimidazole compound that is selectively reduced and covalently bound to intracellular macromolecules in areas of hypoxia (*p*O2 < 10 mm Hg) [15,16]. Preclinical data using pimonidazole in colorectal-cancer-patient-derived xenografts have shown variable amounts of hypoxia (0–40%) within these tumors. Autoradiography showed significant correlation between the presence of ^18^F-FAZA and pimonidazole histochemical staining in tissue sections in these xenograft models. The hypoxic fraction within these lines, estimated using ^18^F-FAZA PET, correlated well with the degree of hypoxia measured by flow cytometry. Furthermore, rectal tumor models with higher baseline ^18^F-FAZA uptake grew faster compared to those with lower baseline ^18^F-FAZA uptake after CRT, suggesting that the more hypoxic tumors responded less to CRT [17]. These preclinical studies suggest that ^18^F-FAZA-PET imaging prior to therapy initiation may serve as an effective clinical biomarker to identify rectal tumor hypoxia. Identification of the presence of hypoxia in rectal cancer prior to neoadjuvant or primary CRT may have prognostic significance and, with the emergence of hypoxia-activated prodrugs, may also have future therapeutic implications [18,19,20,21,22,23,24,25,26,27,28].

The primary goal of this clinical trial was to determine the feasibility of using the ^18^F-FAZA-PET/MR to image primary tumor hypoxia in patients with rectal cancer prior to and following neoadjuvant CRT. The secondary objectives were to determine the optimal method for calculating the hypoxic fraction and to determine whether tumor ^18^F-FAZA uptake changes following CRT.

## 2. Patients and Methods

This institutional ethics review board approved prospective, single-arm pilot study (NCT02624115) including patients with histologically proven locally advanced rectal cancer. Written informed consent was obtained from all participants. The inclusion criteria were 1. Age ≥ 18 years; 2. Histologic proven locally advanced rectal cancer (T3-T4, or N1) based on clinical assessment and standard staging procedures (clinical exam, endoscopy, MRI); 3. Intention to treat with neoadjuvant CRT prior to surgery, according to the current institutional treatment policies; 4. A negative urine or serum pregnancy test within the two-week interval immediately prior to imaging in women of child-bearing age. Exclusion criteria included: 1. Contraindication for MR as per current institutional guidelines; 2. Inability to lie supine for an hour; 3. Pregnancy. Demographic data, including age and gender, histology, and clinical stage were tabulated. Surgical pathology and outcome data (PFS, OS) were tabulated. All eligible study participants received ^18^F-FAZA PET/MR prior to initiation of neoadjuvant concurrent chemotherapy and radiotherapy, as per standard of care and again following completion of CRT prior to surgery. All patients had a follow-up period > 5 years.

### 2.1. PET/MR Imaging

^18^F-FAZA PET/MR imaging of the pelvis was performed using the mMR-Biograph scanner (Siemens, Erlangen, Germany) for 130 ± 18.5 min (mean ± SD) after injection of 469 ± 83 MBq of ^18^F-FAZA. PET had a 25.8 cm axial field of view in the z-direction, with 1 bed position to cover the entire pelvis, with acquisition time of 20 min/bed. PET image reconstruction was performed using conventional ordinary Poisson-ordered subset maximum expectation maximization (OP-OSEM) with 3 iterations, 21 subsets, and FWHM = 4.5 filtering. To assess feasibility of left ventricle as a reference for calculating hypoxic fraction, an additional single bed position over the chest was obtained to include the heart, with acquisition time of 5 min/bed position. MRI-based attenuation correction maps were generated from a 2-point Dixon gradient-echo sequence in the coronal plane, as described elsewhere [29]. MR was performed with a vendor-standard torso phased array surface coil. The protocol included 1-T_2_-weighted fast-spin echo sequences in three planes (sagittal, coronal, and axial), TE/TR = 103/4000, resolution = 0.625 × 0.625 × 4 mm^3^; 2-high-resolution oblique T_2_-weighted FSE, TE/TR = 80/4930, resolution = 0.625 × 0.625 × 3 mm^3^; 3-axial T_1_-weighted images, axial diffusion weighted image sequence (b-value = 0, 100 and 800 s/mm^2^); and 4-multiphasic gadolinium contrast-enhanced T_1_-weighted imaging sequences, including late arterial, venous, and delayed phases (TE/TR = 1.59/4.44, resolution = 0.75 × 0.75 × 4 mm^3^). The oblique T_2_-weighted imaging sequence was angled perpendicular to the long axis of the rectal tumor. Patients received an intravenous bolus of gadobutrol (Gadovist; Bayer AG, Leverkusen, Germany) at the recommended dose (0.1 mL/kg of body weight). To reduce colonic motility, all patients received 20 mg of antiperistaltic agent hyoscine butylbromide (Buscopan, Boehringer Ingelheim, Ingelheim, Germany) 30 min before the examination.

### 2.2. Data Interpretation and Calculation of Hypoxic Fraction

PET in general, and ^18^F-FAZA PET in particular, lack anatomical resolution, limiting assessment of tracer uptake in the tumor on PET images. PET/MR allows for simultaneous acquisition of PET and MR data, enabling segmentation of rectal tumors on MR and using the derived mask to segment the tumor volume on the ^18^F-FAZA PET images. For this purpose, T_2_-weighted MR images of the rectal tumor were used for tumor delineation and segmentation. An experienced abdominal radiologist, blinded to the PET data, contoured the entire primary rectal tumor on MR and the gluteus maximus muscles bilaterally on 6 consecutive slices [30]. The MR-derived tumor contours were propagated onto the PET images. Chronic hypoxia SUV distribution has been previously shown to best fit a Gaussian distribution [31]. To correct for spillover of radiotracer signal from the bladder, which may result in positively skewed tracer uptake distribution in tumors adjacent to the bladder, a systematic approach was used to remove voxels in the tumor contributing to higher uptake than an assumed Gaussian distribution in a method previously described [30].

**Figure 1:**
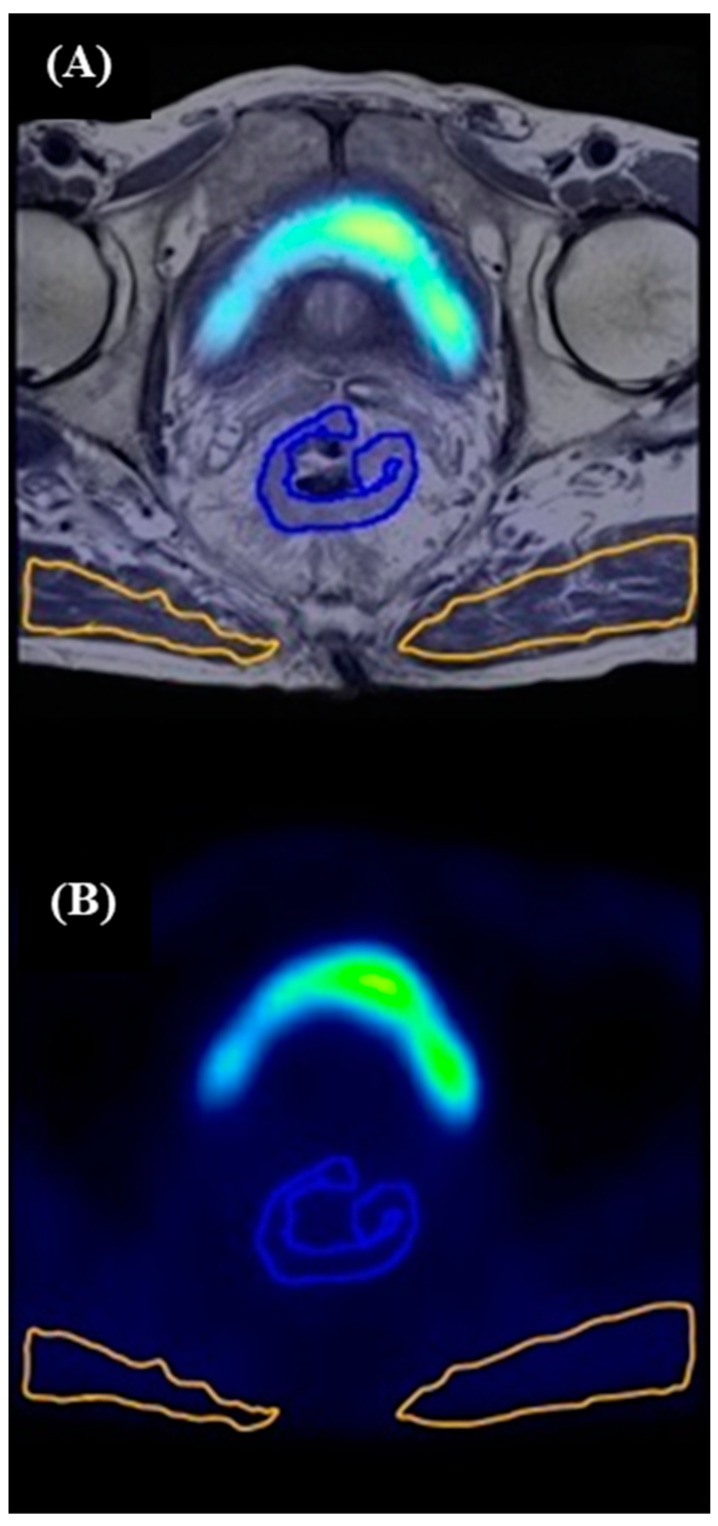
Segmentation method guided with T_2_-weighted MR image fused with ^18^FAZA PET image (**A**) and corresponding ROI in ^18^F-FAZA PET image alone (**B**). Segmentation method. Axial T_2_-weighted MR image at the level of mid-rectum with tumor segmented (blue contour) and gluteus maximum muscle segmented bilaterally (orange contour).

To determine the hypoxic volume or hypoxic fraction, quantitative measures of hypoxia within a tumor (a threshold based on a reference tissue) are needed. Blood pool activity on ^18^F-FAZA PET has been previously validated as a surrogate for blood sampling in a cohort of patients with cervical cancer. In that study, blood samples were counted in a gamma well counter that was cross-calibrated to the scanner, allowing generation of blood sample-derived SUV. Image-derived left ventricular SUVmean and blood sample-derived SUVmean were highly correlated (Pearson correlation coefficient R = 0.996) [31]. We calculated HF using methodology adapted from Mortensen et al. [30]. In the current study, we interrogated the use of the following references: blood pool as measured in the iliac artery (=BP), blood pool as measured in the left ventricle (=LV), and muscle as measured in the gluteus maximus muscle (contracted by 4 mm to avoid partial volume effects = GMc) (Figure 1). Each tumor voxel was defined as either oxic or hypoxic using thresholds of ×1.0 or ×1.2 SUVmean in the references BP and LV or SUVmean GMc +3SD, as previously described [32,33]. The hypoxic fraction (HF) was defined as the ratio of the hypoxic voxels to the total number of voxels in the tumor.

### 2.3. Statistical Analysis

To establish the suitable reference that can represent HF, the tumor (SUVmax)-to-reference (SUVmean) ratio (TRR) for each reference was compared to HF using Pearson correlation testing. The test was performed on data at both thresholds, ×1.0 and ×1.2.

## 3. Results

**Figure 2:**
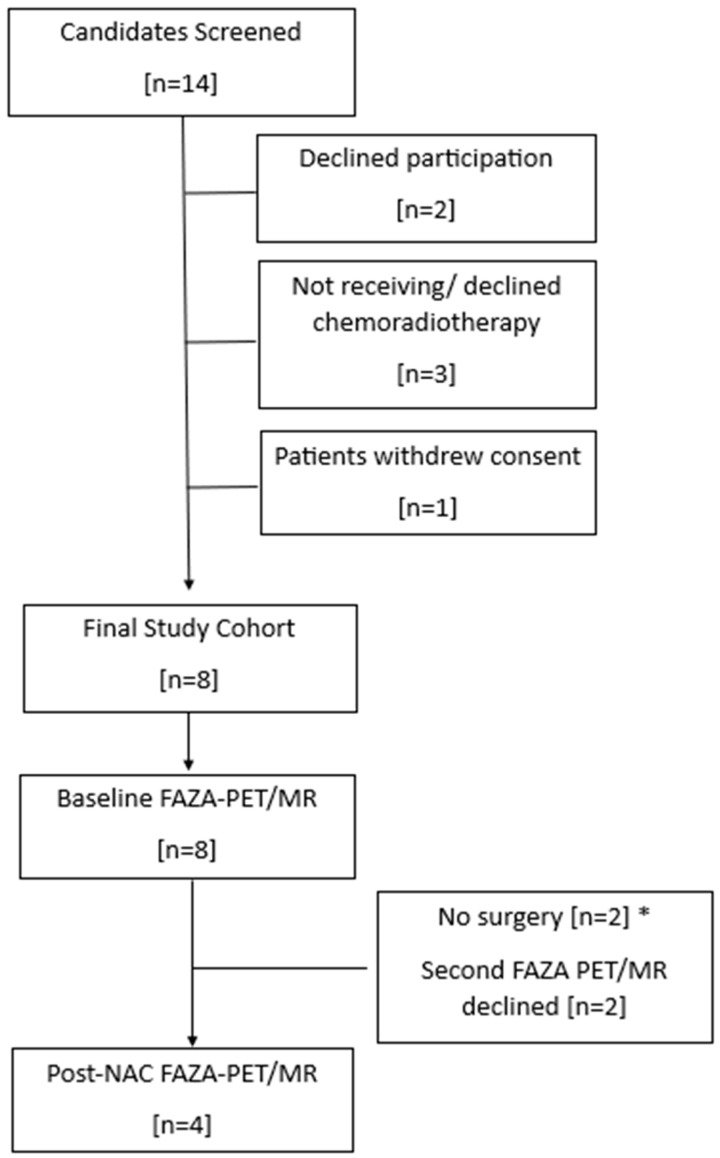
Patient flowchart. NAC = neoadjuvant chemoradiotherapy. * Patient did not undergo surgery and were excluded from follow-up.

There were 14 patients screened for the trial over a 26-month period (see patient flowchart in Figure 2), with 8 consenting patients with locally advanced adenocarcinoma of the rectum who underwent baseline ^18^F-FAZA PET/MR. All patients were men with a mean age of 64.3 years (range: 45–76). Of these, four patients also completed ^18^F-FAZA PET/MR following chemoradiotherapy. Demographic data, including clinical and surgical stage, pathology regression grade, and clinical outcome data, are depicted in Table 1. In Table 2, tumor volume, SUVmax, and SUVmean (±SD) for tumor and reference regions are presented. Following chemoradiotherapy, tumor volume decreased by an average of 69.8% (from baseline to follow-up).

### 3.1. Hypoxic Fractions at Baseline

The distribution of HFs for each of the references and thresholds used are displayed in Table 3 for baseline (n = 8) and following nCRT in Table 4 (n = 4). For GMc (+3SD) and for BP and LV with a threshold of 1.0, the baseline HFs were (median; range) 16.6% (2.4–33.8), 36.8% (0.3–72.9), and 30.7% (0.8–55.5), respectively. For a threshold of ×1.2, the baseline HFs for BP and LV were (median; range) 10.4% (0–47.6), and 4.3% (0–20.1%), respectively.

### 3.2. Comparison of Reference Tissues

**Figure 3:**
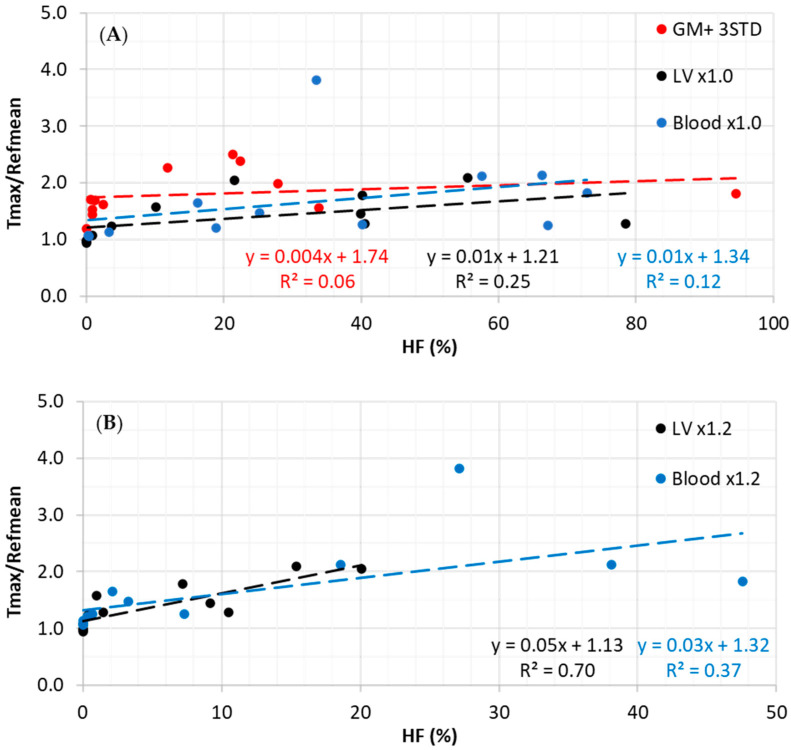
Correlation between TRR and hypoxic fraction using threshold of ×1.0 (**A**) and a threshold of ×1.2 (**B**) for both baseline and following neoadjuvant chemoradiotherapy cases.

For baseline and following neoadjuvant chemoradiotherapy, for a threshold of ×1.0, the correlation coefficients between HF and TRR for GMc (muscle), BP, and LV as references were 0.241, 0.344, and 0.499, respectively (Figure 3A). For a threshold of ×1.2, the correlation coefficients between HF and TRR for BP and LV as references were 0.611 and 0.838, respectively (Figure 3B).

At the threshold of ×1.0, the Pearson correlation between TRR and HF was the strongest for the LV reference (R = 0.499; *p*-value = 0.09) over other references and followed by the BP reference (R = 0.344; *p*-value = 0.29). Meanwhile, a weak correlation between HF and TRR for GMc was measured to be R = 0.241, *p*-value = 0.45. For threshold ×1.2, the correlation for LV was statistically significantly strong (R = 0.837; *p*-value = 0.0007) in comparison to a moderate correlation for BP reference (R = 0.61; *p*-value = 0.035). These results suggest that at ×1.2 threshold, the LV as a reference is more stable to represent the HF.

### 3.3. Change in ^18^F-FAZA Uptake and Tumor HF Following Neoadjuvant Chemoradiotherapy

The HF for the various thresholds following nCRT is presented in Table 4, and the change in tumor SUVmax/reference SUVmean using the various reference regions following nCRT is depicted in Table 5. Using BP or LV as references, there were two patients whose HF decreased after neoadjuvant therapy and two whose HF increased. Both patients whose HF increased had complete or near-complete pathologic response to therapy (grade 0 or 1).

## 4. Discussion

In patients with locally advanced rectal cancer prior to neoadjuvant therapy, ^18^F-FAZA PET/MR shows variable degrees of tumor hypoxia. This observation is based on comparison of radiotracer uptake within tumors to reference tissues, blood, or muscle. Using the optimal reference for calculating the hypoxic fraction is crucial. Although blood pool has been previously suggested to be the most reliable, regional skeletal muscle has been used by researchers as a reference due to ease of sampling [34]. In our sample, the most reliable reference standard appeared to be blood with measurements obtained by placing a region of interest over the iliac artery or the left ventricle. As previously observed by Han et al. [30,34], the LV seemed the most reliable reference, with a correlation coefficient of 0.499 and 0.838 for a threshold of ×1.0 and ×1.2, respectively. Blood pool activity as obtained from the iliac artery appears to be a suitable alternative, especially if scan time constraints limit sampling of the left ventricle. The threshold used to define hypoxia is important and directly impacts the defined hypoxic volume. The higher the threshold, the lower the correlation coefficients with HF, suggesting decreased reliability. Correlation with an external reference standard would be needed to validate the suggested threshold.

A prior study using pimonidazole staining to identify hypoxia in pathology specimens in 20 patients with colorectal tumors showed variable hypoxia with HF ranging from 2.2 to 37.8% [9]. The only prior study to report the use of ^18^F-FAZA in rectal cancer was reported by Havelund et al. using a PET/CT scanner [35]. The authors concluded that hypoxia in rectal cancer at baseline can be assessed using ^18^F-FAZA. They quantified the uptake of radiotracer in the tumor after correcting for signal spill from bladder using the ratio between the tumor SUVmax and the normal intestinal SUVmean. Similarly, in our patient cohort using ^18^F FAZA PET/MR, there was variable hypoxia in locally advanced rectal adenocarcinomas. The use of ^18^F-FAZA PET/MR allowed us to measure and characterize the change in the tumor volume following radiotherapy (Table 2). We assessed various thresholds. When using LV with a threshold of ×1.0 as reference, HF ranged from <1% to 55.5% (median, 30.7%). A further observation from our study was variable change in hypoxia following nCRT. Interestingly, two patients with increased hypoxia following therapy had complete or near-complete pathologic response to therapy, suggesting that the hypoxia imaged following radiotherapy may be due to radiotherapy-induced inflammation (radiation-induced proctitis) rather than residual hypoxic tumor. Chronic inflammation, including radiation-induced proctitis, may result in fibrosis, obliterative endarteritis, and tissue hypoxia [36]. These findings suggest that following therapy, hypoxia measured on PET needs to be interpreted with caution and in conjunction with tumor regression. Hypoxia imaging with PET offers several advantages. It is minimally invasive, reproducible, and, as we have shown, can be repeated to assess tumor hypoxia before and after a therapeutic intervention. The advantage of PET/MR is the ability to simultaneously obtain PET and MR data enabling accurate delineation of tumor and radiotracer uptake within the tumor. This is especially important at sites which may alter their morphology and location in a short period of time (e.g., due to peristalsis), such as the rectum. MR also enables assessment of morphological response to nCRT, which appears to also be important in the interpretation of PET-measured hypoxia after radiotherapy.

Aside from prognostication, identification of hypoxia within LARC may have therapeutic implications. Hypoxia-activated prodrugs, currently under research, are compounds which under hypoxic conditions can be selectively reduced by specific reductases to form cytotoxic agents that target hypoxic tumor cells, with little toxicity to normal tissues [37]. Preclinical studies using patient-derived colorectal cancer xenografts have shown that ^18^F-FAZA PET can be used as a biomarker for hypoxia to identify patients that would benefit most from addition of hypoxia-activated prodrugs [17]. The researchers showed that the addition of evofosfamide, a hypoxia-activated prodrug, to chemoradiotherapy better inhibited tumor growth and decreased the fraction of cancer-initiating cells [17]. Further trials in humans are needed to confirm the clinical value of this potential novel therapeutic approach in terms of patient outcomes.

There are several limitations to our study. First, the study population was limited, especially the subgroup of patients with pre- and post-CRT data. This was due to difficulty in recruiting patients with a newly diagnosed malignancy prior to a prolonged multimodality treatment protocol who were agreeable to commit to two additional research imaging sessions. Despite the limited patient population, we were able to confirm the prior observations of variable hypoxia in rectal tumors as shown in pimonidazole staining of clinical pathology specimens and from patient-derived human xenografts [17,35]. Second, we did not have an objective reference standard such as pimonidazole staining in tumors or intraoperative O_2_ intratumor measurements. Nonetheless, our data showing variability of hypoxia in tumors prior to therapy are in line with a previous series using pimonidazole staining in pathology specimens. Third, although we have shown a wide variability in HF in locally advanced rectal cancers, the clinically relevant threshold of HF as a predictor of poor response to CRT remains uncertain. This would be difficult to determine even in larger scale prospective trials, as there are multiple variables that impact therapy response and patient outcomes.

## 5. Conclusions

In conclusion, non-invasive imaging of hypoxia in locally advanced rectal cancers with ^18^F-FAZA PET/MR imaging is feasible. Blood pool as measured in the left ventricle is the most reliable reference for calculating the HF. There is a wide range of HFs and variable change in HF before and after neoadjuvant CRT. The use of ^18^F-FAZA PET/MR imaging should be considered in future trials assessing clinical utility of hypoxia-targeting drugs in patients with rectal cancer.

## Figures and Tables

**Table 1:** Demographic and outcome data.

Patient ID	Age	Gender	Clinical Stage	Pathology Stage	TRG	Follow-Up
4	69	M	cT3N1M0	ypT3N1c	Grade 3	LR; 14 months
5	45	M	cT2N1M0	ypT2pN0	Grade 1	NED; 5 years
6	47	M	cT3N1M0	ypT3pN1a	Grade 2	NED; 5 years
7	74	M	cT3N1M1	ypT3N1a	N/A	NED; 5 years
8	76	M	cT3N2M0	N/A	N/A	N/A
9	70	M	cT3N1M0	ypT2pN0	N/A	NED; 5 years
10	69	M	cT2N1M0	ypT0	Grade 0	NED; 5 years
11	64	M	cT3N2M1	N/A	N/A	PD; 3 months

TRG = tumor regression grade (AJCC): grade 0 = no residual disease; grade 1 = near complete response/minimal residual disease; grade 2 = moderate response (residual cancer outgrown by fibrosis); grade 3 = poor response, minimal regression. LR = local recurrence; NED = no evidence of disease recurrence; PD = progressive disease (systemic).

**Table 2:** Measured tumor volume, SUVmax, and SUVmean and standard deviation (SD) for references and tumor at baseline and following neoadjuvant chemoradiotherapy.

		Volume (mL)	SUVmax	SUVmean ± SD			
		Tumor		Tumor	GMc	BP	LV
Baseline	P4	41.43	1.96	0.94 ± 0.19	0.86 ± 0.10	0.92 ± 0.09	0.93 ± 0.10
	P5	20.35	1.30	0.55 ± 0.23	0.77 ± 0.11	1.22 ± 0.18	1.37 ± 0.17
	P6	22.88	1.43	0.74 ± 0.17	0.84 ± 0.11	0.67 ± 0.06	0.80 ± 0.11
	P7	16.70	2.42	1.13 ± 0.34	1.01 ± 0.13	1.46 ± 0.11	1.54 ± 0.17
	P8	18.80	2.88	3.14 ± 2.56	1.18 ± 0.19	1.21 ± 0.22	1.43 ± 0.18
	P9	2.03	1.45	1.02 ± 0.25	0.93 ± 0.09	1.14 ± 0.20	1.13 ± 0.14
	P10	6.05	1.45	0.88 ± 0.17	0.89 ± 0.12	1.35 ± 0.20	1.34 ± 0.22
	P11	60.96	2.31	1.48 ± 0.31	1.16 ± 0.18	1.26 ± 0.17	1.59 ± 0.17
Follow-up	P4	12.80	0.95	0.65 ± 0.10	0.81 ± 0.09	0.84 ± 0.10	0.99 ± 0.11
	P5	7.19	1.34	0.96 ± 0.16	0.88 ± 0.15	1.11 ± 0.16	1.36 ± 0.16
	P6	4.67	1.56	0.97 ± 0.15	1.09 ± 0.12	1.06 ± 0.08	1.26 ± 0.12
	P10	2.07	1.66	1.39 ± 0.13	0.92 ± 0.09	1.33 ± 0.25	1.29 ± 0.13

References: BP = blood pool; GMc = contracted gluteus maximus muscle; LV = left ventricle; SD = standard deviation.

**Table 3:** Distribution of baseline hypoxic fraction (%).

Threshold SUVmean	Reference	Patient Number							
		P4	P5	P6	P7	P8	P9	P10	P11
+3SD	GMc	11.8	1.2	0.5	22.4	21.3	33.8	2.4	27.9
×1.0	BP	57.6	0.3	66.3	16.2	33.4	40.2	0.3	72.9
	LV	55.5	0	40.2	10.1	21.5	40.5	0.8	39.9
×1.2	BP	18.6	0	38.1	2.1	27.0	0.7	0	47.6
	LV	15.4	0	7.2	1.0	20.1	1.4	0	9.2

References: BP = blood pool; GMc = contracted gluteus maximus muscle; LV = left ventricle. Threshold: ×1.0 or ×1.2 of reference SUVmean for BP and LV or GMc SUVmean +3SD.

**Table 4:** Distribution of hypoxic fraction (%) following neoadjuvant chemoradiotherapy.

Threshold SUVmean	Reference	Patient Number			
		P4	P5	P6	P10
+3SD	GMc	0	0.8	0.8	94.6
×1.0	BP	3.3	18.9	25.2	67.2
	LV	0	0	3.6	78.5
×1.2	BP	0.3	3.3	7.3	0
	LV	0	0	0.4	10.5

GMc = contracted gluteus maximus; BP = blood pool; LV = left ventricle; SD = standard deviation. Thresholds of ×1.0 and ×1.2 SUVmean for BP or LV and SUVmean +3SD for GMc.

**Table 5:** Relative change in tumor SUVmax/reference SUVmean (%) following neoadjuvant chemoradiotherapy for various references.

Threshold SUVmean	Reference	Patient Number			
		P4	P5	P6	P10
+3SD	GMc	−47.7	−9.6	−15.9	11.3
×1.0	BP	−46.6	13.7	−31	15.8
	LV	−54	4.5	−30.5	18.9

GMc = contracted gluteus maximus; BP = blood pool; LV = left ventricle; SD = standard deviation. Threshold of ×1.0 SUVmean for BP or LV and SUVmean +3SD for GMc.

## Data Availability

The data presented in this study are available on request from the corresponding author.
